# Editorial: Isolation, identification, characterization, and utilization of beneficial microbes for crop improvement

**DOI:** 10.3389/fpls.2026.1792485

**Published:** 2026-03-02

**Authors:** Jyostna Devi Mura, Amita Kaundal, Tripti Vashisth, Padma Nimmakayala

**Affiliations:** 1 Vegetable Crops Research Unit, US Department of Agriculture (USDA)—Agricultural Research Service (ARS), Madison, WI, United States; 2 College of Agriculture and Applied Sciences, Utah State University, Logan, UT, United States; 3 Citrus Research and Education Center, University of Florida, Lake Alfred, FL, United States; 4 Gus R. Douglass Institute and Department of Biology, West Virginia State University, Institute, WV, United States

**Keywords:** beneficial microbes, biodiversity, climate resilience, crop improvement, plant microbiome

Global food production is increasingly constrained by climate variability, soil degradation, and long-term reliance on chemical fertilizers and pesticides. In this context, beneficial microbes have emerged as practical and sustainable tools for improving crop productivity while reducing environmental impacts. These microorganisms influence plant health through diverse mechanisms, including nutrient acquisition, disease suppression, and stress tolerance, and are now recognized as integral components of sustainable agricultural systems. The Research Topic “Isolation, Identification, Characterization, and Utilization of Beneficial Microbes for Crop Improvement” brings together studies that advance microbial discovery, functional chacterization and application across diverse agricultural settings.

The five original research articles included in this Research Topic capture the scope and direction of current plant–microbe research. They span multiple systems, including forage preservation, rhizosphere interactions, biological nitrogen fixation, biological control in perennial crops, and genome-guided microbial selection. Collectively, these studies illustrate how microbial solutions can be tailored to specific crops and management goals, while emphasizing the need to connect ecological relevance with functional validation.

Peng et al. examine microbial interventions in forage preservation by evaluating Lactiplantibacillus inoculants combined with sea buckthorn pomace on the fermentation of paper mulberry silage, a protein-rich woody forage that is prone to nutrient loss during ensiling. Their results show lower pH and ammonium nitrogen levels, along with increased lactic and acetic acid production, leading to improved retention of crude protein and water-soluble carbohydrates. The treatment suppresses undesirable microorganisms such as Enterobacter while promoting lactic acid bacteria, resulting in greater feed stability. This work highlights how targeted microbial–substrate combinations can enhance silage quality and extends the relevance of beneficial microbes beyond crop growth to livestock nutrition and feed system sustainability.

In perennial cropping systems, Zanfaño et al. demonstrate the value of agroecosystem-derived microbial diversity for disease management. The authors describe *Trichoderma carraovejensis*, a newly identified fungal species isolated from vineyard environments, and evaluate its activity against grapevine trunk disease pathogens, including *Diplodia seriata*. Morphological, phylogenetic, and functional analyses reveal rapid growth, high conidial production, and strong mycoparasitic activity, with pathogen inhibition reaching up to 34%. The strain’s compatibility with sulfur-based pesticides supports its potential use within existing integrated pest management programs and highlights the advantages of locally adapted biocontrol agents in viticulture.

At the rhizosphere level, Ganesh et al. investigate plant growth-promoting rhizobacteria isolated from the native shrub *Ceanothus velutinus*. The predominantly Pseudomonas strains produce indole-3-acetic acid and exhibit multiple beneficial traits, including nitrogen fixation, phosphate solubilization, and ACC deaminase activity. Inoculation enhances rooting, cutting survival, and biomass accumulation in both the host plant and Arabidopsis thaliana, while reducing the effects of drought stress. These findings underscore the role of phytohormone-mediated and nutrient-related processes in microbial-driven plant growth and point to potential applications in plant propagation and cultivation under water-limited conditions.

Addressing Nutrient use efficiency, Aasfar et al., evaluate diazotrophic bacteria such as *Rhodotorula mucilaginosa* and *Arthrobacter* sp. for their effects on wheat grown under different nitrogen and phosphorus regimes. The tested strains enhance biological nitrogen fixation and phosphate solubilization, leading to increases in plant height, chlorophyll content, nutrient uptake, and biomass. Notably, nitrogen fertilizer inputs can be reduced by up to 50% without compromising plant performance. This work highlights the potential of microbial inoculants to reduce fertilizer dependency and improve nutrient management, particularly in phosphorus-limited soils.

Jeon et al. presented a genome-based characterization of a novel *Lelliottia* strain with pronounced plant growth-promoting activity. By combining comparative genomic analyses with functional assays, the authors identify genes associated with phosphate solubilization, nutrient acquisition, and stress tolerance. Application of this strain to tomato plants under phosphorus deficiency results in higher germination rates, increased root and shoot growth, and greater overall biomass. The study demonstrates how genomic approaches can support more efficient screening and selection of microbial candidates for agricultural use.

Overall, the studies in this Research Topic illustrate the diverse and context dependent roles of beneficial microbes in agriculture, from improving silage fermentation and controlling plant diseases to enhancing nutrient availability and stress tolerance. A consistent theme is the importance of sourcing microbes from relevant environments and integrating ecological insight with functional and molecular validation. Aligning microbial traits with specific agronomic needs remains critical for successful application. As agriculture transitions toward biologically based and climate-resilient systems, continued progress will rely on field validation, formulation development, and integration of microbial technologies into existing management practices.

